# Structuprint: a scalable and extensible tool for two-dimensional representation of protein surfaces

**DOI:** 10.1186/s12900-016-0055-7

**Published:** 2016-02-24

**Authors:** Dimitrios Georgios Kontopoulos, Dimitrios Vlachakis, Georgia Tsiliki, Sofia Kossida

**Affiliations:** Department of Life Sciences, Imperial College London, Silwood Park Campus, Ascot, UK; Bioinformatics & Medical Informatics Team, Biomedical Research Foundation, Academy of Athens, Athens, Greece; School of Chemical Engineering, National Technical University of Athens, Athens, Greece; IMGT®, The International ImMunoGeneTics Information System®, Université de Montpellier, Laboratoire d’ImmunoGénétique Moléculaire LIGM, UPR CNRS 1142, Institut de Génétique Humaine, Montpellier, France

**Keywords:** Molecular cartography, Protein surfaces, Visualization, Surface comparison, Structural biology

## Abstract

**Background:**

The term ‘molecular cartography’ encompasses a family of computational methods for two-dimensional transformation of protein structures and analysis of their physicochemical properties. The underlying algorithms comprise multiple manual steps, whereas the few existing implementations typically restrict the user to a very limited set of molecular descriptors.

**Results:**

We present Structuprint, a free standalone software that fully automates the rendering of protein surface maps, given - at the very least - a directory with a PDB file and an amino acid property. The tool comes with a default database of 328 descriptors, which can be extended or substituted by user-provided ones. The core algorithm comprises the generation of a mould of the protein surface, which is subsequently converted to a sphere and mapped to two dimensions, using the Miller cylindrical projection. Structuprint is partly optimized for multicore computers, making the rendering of animations of entire molecular dynamics simulations feasible.

**Conclusions:**

Structuprint is an efficient application, implementing a molecular cartography algorithm for protein surfaces. According to the results of a benchmark, its memory requirements and execution time are reasonable, allowing it to run even on low-end personal computers. We believe that it will be of use - primarily but not exclusively - to structural biologists and computational biochemists.

**Electronic supplementary material:**

The online version of this article (doi:10.1186/s12900-016-0055-7) contains supplementary material, which is available to authorized users.

## Background

Over the last two decades, the growth rate of the Protein Data Bank has been exponential. As structural data for biomolecules are increasingly made available, the study of homologous proteins can be performed not only at the level of sequence, but also at the level of three-dimensional structure. This has led to the development of numerous sophisticated methods, concerning, among others, the analysis of structural evolution [1] and the structure-based design of new drugs [2].

For the comparison of protein surfaces in particular, a family of methods is based on the reduction of the dimensionality of the system. The concept of projecting a three-dimensional protein structure to two dimensions was first introduced by Fanning et al. under the term ‘molecular cartography’ [3]. They presented this notion as a novel method for studying the entire surface of a protein, emphasizing on the topography of antigenic sites. It involved conversion of the protein structure into a triaxial ellipsoid, followed by its transformation into a graticule (a latitude/longitude grid). Pawłowski and Godzik later expanded on this approach by annotating protein surface maps according to the physicochemical properties of the exposed residues (e.g., charge or hydrophobicity), as a means to compare evolutionarily related proteins [4].

Even though a number of modifications to the aforementioned methodologies for two-dimensional protein representation have been proposed [5–7], molecular cartography has not found much use in the literature. This may be partly due to the significant amount of effort that is required to manually convert the atomic coordinates of a PDB file first into a spherical structure and then into a map. Visualizing the distribution of a particular physicochemical property on the surface further increases the complexity and the overall approach becomes increasingly tedious. A few applications that implement molecular cartography algorithms are available (SURF’S UP! [8], PST [9], Udock [10]), but the range of supported physicochemical descriptors for visualization is typically limited to charge and hydrophobicity. Integrating other predictors is either unfeasible or not straightforward for the end user, creating an obstacle for specialized analyses. Moreover, an application that harnesses the power of multiprocessor systems to simultaneously render multiple protein surface maps is not to this day available. This would be very useful, for example, when visualizing entire molecular dynamics simulations or comparing the members of a large protein family.

**Fig. 1:**
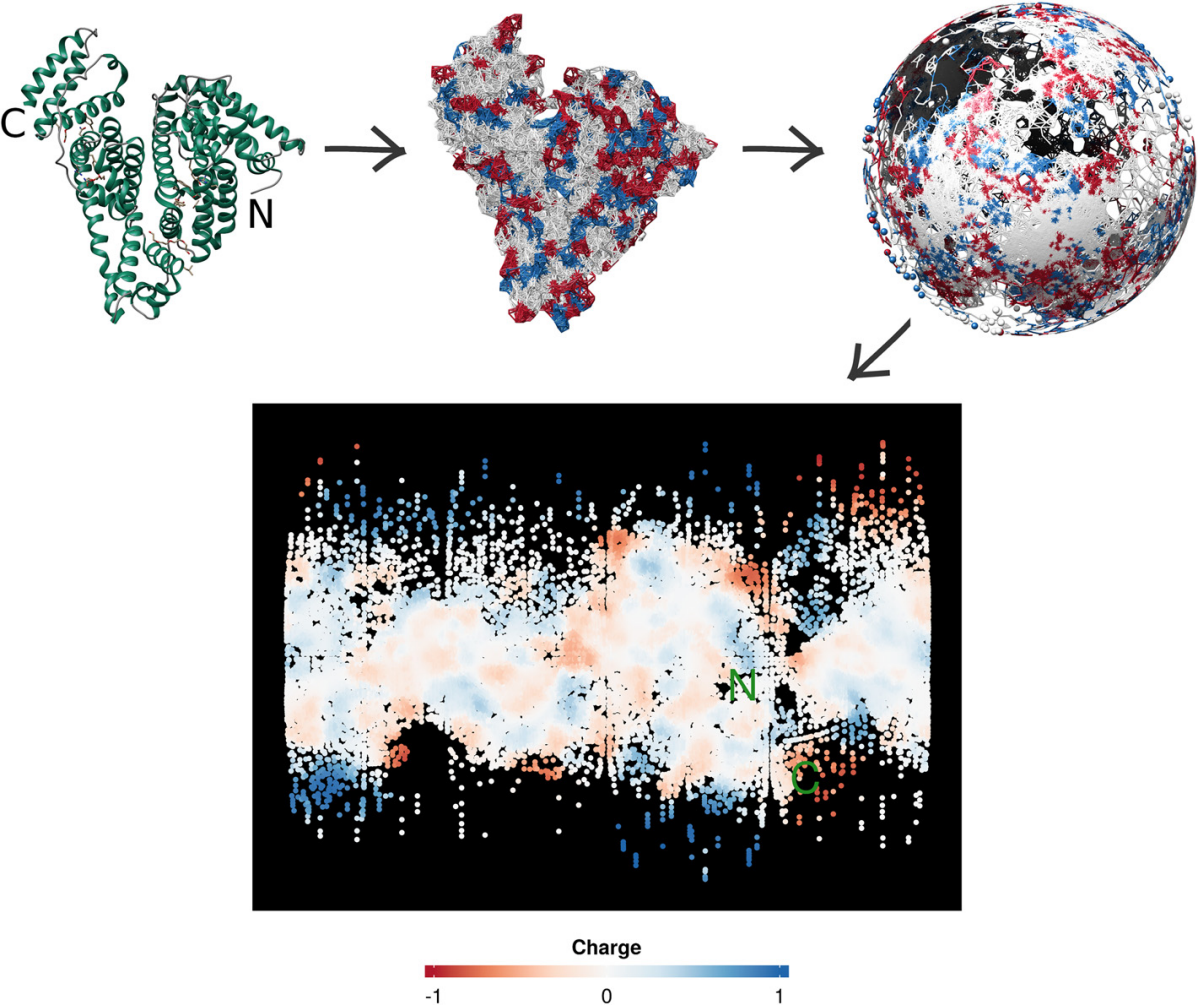
The main steps of the algorithm executed by Structuprint. Here, a mould of the surface of the 3D structure of the leporine serum albumin ([PDB: 4F5V]) is first generated. The property values (e.g., charge) of the amino acids below the mould are retained. Then, the dummy atoms consisting the mould are mapped onto a sphere. Finally, the sphere is projected onto a map using the Miller cylindrical transformation and a smoothing of the property values is performed. The elements of the upper half of the figure were rendered with UCSF Chimera

To fill these gaps, we introduce Structuprint, a new tool for visualization of protein surfaces in two dimensions. Its name is a combination of the terms ‘structure’ and ‘fingerprint’, alluding to the fingerprint-like figures that it generates (see Fig. 1 for an example). Structuprint can produce single 2D maps starting from a PDB file, or GIF animations from multiple files. It is designed with a focus on scalability and extensibility. The tool can utilize multiple CPU cores on GNU/Linux and OS X machines and can easily incorporate any physicochemical predictors provided by the user, other than those in its own default set. The following sections describe the design choices behind its algorithm, present the results from a benchmark and show three characteristic examples of use.

## Implementation

### Amino acid properties database

Values for 328 properties/descriptors were calculated for the 20 common amino acids with MOE 2010.10 [11] and were stored within an SQLite database. In particular, the database contains 11 categories of descriptors: i) 33 adjacency and distance matrix descriptors [12–16] (e.g., Balaban’s connectivity topological index [14]); ii) 41 atom/bond count descriptors [17, 18] (e.g., the number of double bonds); iii) 18 conformation dependent charge descriptors [19] (e.g., the water accessible surface area of polar atoms); iv) the 16 Kier and Hall connectivity and kappa shape indices [20, 21] (e.g., the Zagreb index); v) 21 MOPAC descriptors [22] (e.g., the ionization potential); vi) 48 partial charge descriptors (e.g., the total positive partial charge); vii) 12 pharmacophore feature descriptors (e.g., the number of hydrophobic atoms); viii) 11 potential energy descriptors (e.g., the solvation energy); ix) 16 physical properties [18, 23–27] (e.g., the molecular weight); x) 18 subdivided surface areas; xi) 94 surface area, volume, and shape descriptors (e.g., globularity). A detailed explanation of each descriptor is provided in the properties codebook which accompanies the tool. By drawing values from this database, Structuprint can visualize the distribution of a property across protein surfaces. Users can extend it by adding measurements for more chemical components or provide their own custom SQLite database in order to incorporate novel descriptors.

### Algorithm

#### Generation of a mould of the surface of a protein

The main steps of the algorithm implemented by Structuprint are shown in Fig. 1. The tool first produces a mould of the protein structure’s surface in two steps. The structure is initially placed within a 3D grid with cell dimensions of 1 × 1 × 1 Å. Then, one dummy atom is inserted in each empty grid cell that neighbours a single protein atom. This process was previously described by Vlachakis et al. [28] and is extended here, with dummy atoms being assigned the identity of the amino acid to which their neighbouring protein atom belongs. This results to a quite accurate approximation of the underlying protein surface at the level of residue atoms.

#### Transformation of the mould into a sphere

The next step involves the conversion of the dummy atoms mould to a sphere. To this end, the algorithm calculates the coordinates of the centre of mass of the mould **c** - i.e., the average position of all atoms -, and the maximum distance of any atom **v**_***i***_ from the centre of mass (*radius*):

1 $$ \mathbf{c}=\left({x}_c,\kern0.75em {y}_c,\kern0.75em {z}_c\right)=\left(\frac{{\displaystyle {\sum}_{i=1}^n}{x}_i}{n},\kern0.75em \frac{{\displaystyle {\sum}_{i=1}^n}{y}_i}{n},\kern0.75em \frac{{\displaystyle {\sum}_{i=1}^n}{z}_i}{n}\right) $$

2 $$ radius=\underset{1\le i\le n}{ \max}\sqrt{{\left({x}_i-{x}_c\right)}^2+{\left({y}_i-{y}_c\right)}^2+{\left({z}_i-{z}_c\right)}^2\ } $$

The coordinates of each atom are normalized with respect to the centre of mass:

3 $$ {\mathbf{v}}_{\boldsymbol{i}}^{\boldsymbol{\hbox{'}}}=\left({x}_i^{\hbox{'}},\kern0.75em {y}_i^{\hbox{'}},\kern0.75em {z}_i^{\hbox{'}}\right) = \left({x}_i-{x}_c,\kern0.75em {y}_i-{y}_c,\kern0.75em {z}_i-{z}_c\right) $$

Then, to transfer the dummy atoms onto the surface of a sphere, each vector **v**_***i***_^'^ is scaled to a length equal to the *radius*:

4 $$ {\mathbf{w}}_{\boldsymbol{i}}=\left({x}_i^{\hbox{'}\hbox{'}},\kern0.75em {y}_i^{\hbox{'}\hbox{'}},\kern0.75em {z}_i^{\hbox{'}\hbox{'}}\right) = \frac{radius}{\sqrt{{x_i^{\hbox{'}}}^2+{y_i^{\hbox{'}}}^2+{z_i^{\hbox{'}}}^2}}\cdot {\mathbf{v}}_{\boldsymbol{i}}^{\boldsymbol{\hbox{'}}} $$

#### Projection of the sphere onto a map

The Cartesian coordinates of each **w**_***i***_ are converted to latitude/longitude values (in units of radians) using the following set of equations:

5 $$ \begin{array}{l} latitud{e}_i={ \tan}^{-1}\frac{z_i^{\hbox{'}\hbox{'}}}{\sqrt{{x_i^{\hbox{'}\hbox{'}}}^2+{y_i^{\hbox{'}\hbox{'}}}^2}}\hfill \\ {} longitud{e}_i={ \tan}^{-1}\frac{y_i^{\hbox{'}\hbox{'}}}{x_i^{\hbox{'}\hbox{'}}}\hfill \end{array} $$

For the two-dimensional projection, several techniques were initially tested (e.g., the sinusoidal projection [29] and the Hammer projection [29, 30]), before deciding on the Miller cylindrical projection [29, 31]:

6 $$ {\mathbf{m}}_{\boldsymbol{i}}=\left( longitud{e}_i,\kern0.75em \frac{5}{4}\cdot \ln \left[ \tan \left(\frac{\pi }{4}+\frac{2}{5}\cdot latitud{e}_i\right)\right]\right) $$

This projection was selected on the basis of its simplicity and ease of understanding. It is one of the most popular projections in cartography, as it can depict the entirety of the sphere, including the poles. Latitude and longitude lines are parallel and straight. Projection-induced distortion is zero at the equator, increases gradually towards higher latitudes, and becomes maximal at the poles. This leads to significant overestimation of the distance among atoms at the upper and lower parts of the figure (Fig. 1), similarly to the areal exaggeration of Greenland and Antarctica. Nevertheless, the Miller cylindrical projection introduces less polar distortion than the Mercator projection, on which it is based.

#### Map smoothing

The previous step resulted in a map of the protein surface with data points coloured by a property of choice. However, this ‘primary’ map is not suitable for detecting areas with an overall concentration of atoms with high or low property values, which is one of the main benefits of this cartographic approach. For instance, a small area with both negatively and positively charged residue atoms would not appear as almost neutrally charged, but as a tiny dipole. To prevent the appearance of small ‘hot spots’ and redistribute the property values among neighbouring data points, the algorithm includes a smoothing step. The map is iteratively divided in grid squares of varying dimensions, from 0.001° × 0.001° to 0.5° × 0.5°, with a step increase of 0.001°. In each iteration of this process, grid cells are assigned the average value of all data points within them. Finally, the value of every data point is defined as the average value of its corresponding grid cell across all iterations. This smoothing method ensures that areas with pronounced accumulation of high or low values are easily discernible from those with a mixed population.

### User interfaces

The default interface of Structuprint is a cross-platform, command-line interface (CLI). It consists of two executables: structuprint_frame and structuprint. The structuprint_frame executable produces a TIFF figure from a single input PDB file, using the R package ggplot2 [32] for plotting. The structuprint executable is responsible for processing multiple superimposed PDB files - either serially or in a parallel manner -, generating a TIFF figure per input file and a final GIF animation, rendered with the Imager Perl module [33]. Most parameters of the underlying algorithms can be modified by the user, such as the delay between animation frames, the background colour, and the appearance of ID numbers on final figures. A full descriptive list of the available parameters for both executables can be found in Structuprint’s manual, distributed along with the application and also available from its website.

**Fig. 2:**
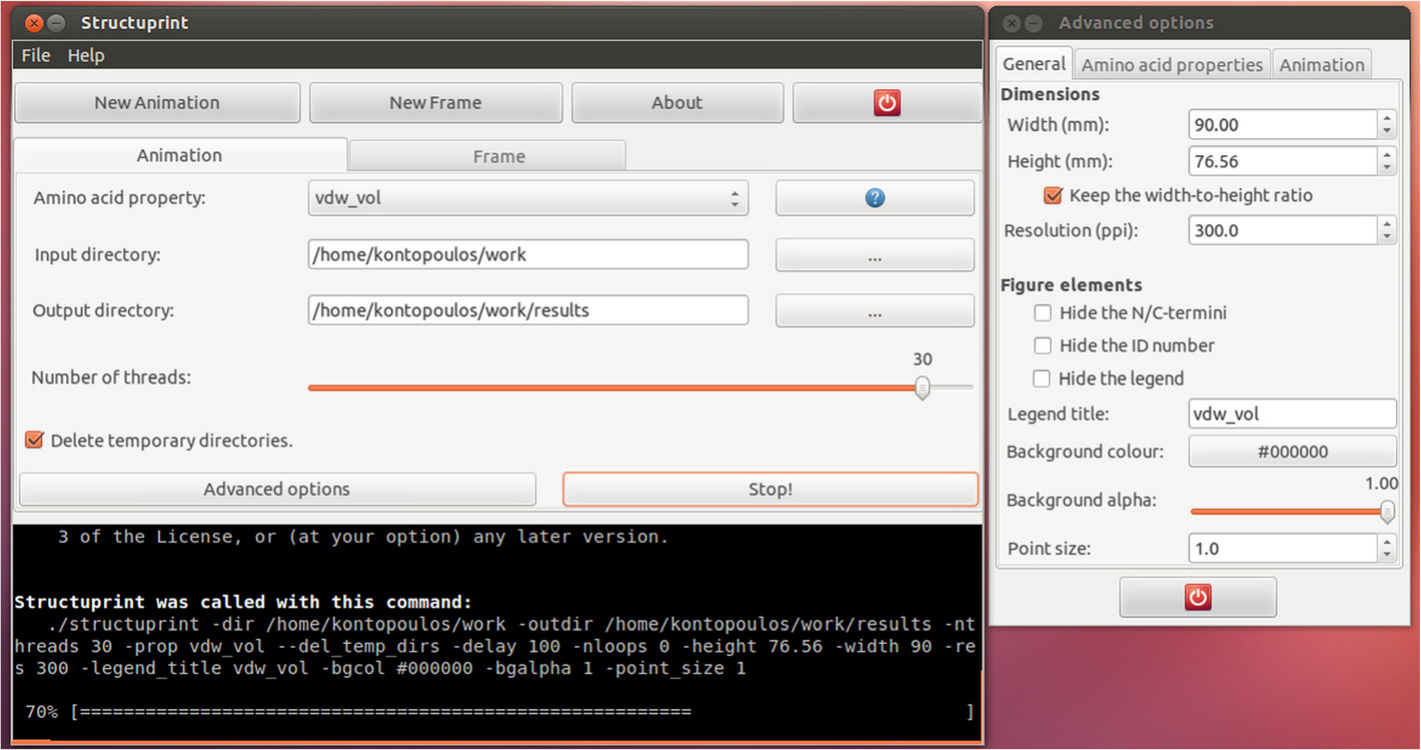
Structuprint’s Graphical User Interface. The main window is split between two tabs for preparation of 1) animations and 2) single static maps. The default parameters of the algorithm can be modified using the ‘Advanced options’ popup window. When Structuprint is rendering a figure, its progress is shown in a temporary terminal

Other than the CLI, Structuprint also comes with a Graphical User Interface (GUI), available by default only on GNU/Linux systems. The GUI is built with the Gtk2 toolkit and offers a user-friendly interface to all the command line arguments and options. As an example of its capabilities, in Fig. 2 Structuprint’s GUI is producing an animation on a multiprocessor machine using 30 cores.

### Parallelism

On Unix-like systems (e.g., GNU/Linux, OS X), Structuprint supports task parallelism when generating animations. Using the Parallel::ForkManager Perl module [34], Structuprint can take advantage of multiple CPU cores by assigning each input PDB file to a different processor. The simultaneous rendering of multiple individual frames considerably reduces the total execution time, allowing for visualization of entire molecular dynamics simulations within a reasonable time frame.

## Results and discussion

### Benchmark

**Fig. 3:**
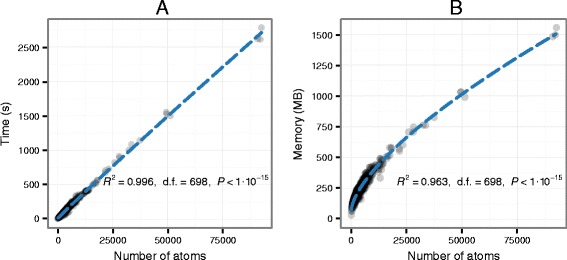
Execution time (**a**) and memory consumption (**b**) of Structuprint, as a function of the atom count (n). The runtime complexity is O(n), whereas the memory complexity is $\mathrm{O}\left(\sqrt{\mathrm{n}}\right)$. The uneven distribution of atom counts reflects the composition of the Protein Data Bank. As of March 2015, ~99 % of entries in the PDB had an atom count of 61,000 or less, with the overall mean being 9,006 atoms

To understand how execution time and memory consumption scale with the number of atoms in an input PDB file, we ran Structuprint against 700 randomly selected structures from the Protein Data Bank (Additional file 1). For simplification purposes, multi-model PDB entries were excluded, as a large proportion of the atoms would overlap in 3D space, being essentially indistinguishable. The benchmark was performed on a GNU/Linux system with an Intel Xeon E5-1650 v2 CPU at 3.50 GHz and 31.4 GB of memory. Structuprint was launched 10 times per PDB file and the execution time was measured as the median time for completion. Memory usage was measured similarly. We then performed linear regressions using execution time and memory consumption as dependent variables and number of atoms as the independent variable. In both regressions, we applied a Box-Cox transformation [35] to the dependent variable to ensure that the residuals were normally distributed. The final fitted models are shown in Fig. 3. Execution time increases linearly with the number of atoms, whereas memory consumption only increases with the square root of the atom count. For example, on the aforementioned system it took 88 seconds and 211 MB of RAM to generate a Structuprint figure for a relatively small protein with 2,461 atoms ([PDB:1YLP]).

### Examples of usage

To illustrate the utility of this tool, we present three different examples of usage in this section. Two-dimensional visualization with Structuprint enhances the representation of protein surfaces and facilitates the interpretation of the results in all three cases.

#### Visualization of molecular dynamics simulations

A seldom explored application of molecular cartography involves the generation of 2D animations from a series of PDB files. Here, we visualized a portion of a folding simulation of a variant of the chicken villin headpiece subdomain (HP-35 NleNle) from the Folding@Home project [36]. The part of the input simulation was 50 ps long, with one frame being extracted every 0.25 ps. Each frame was structurally superimposed to the previous one with UCSF Chimera’s MatchMaker tool [37]. Then, two separate animations were produced: one of the simulation frames in ribbon representation and one of the corresponding 2D maps, with the topological polar surface area - a measure of polarity - as the property of choice. For comparison purposes, these two animations are jointly shown in Additional file 2. This approach simplifies the detection of conformational changes during the course of the simulation, along with fluctuations in the distribution of physicochemical variables.

#### Depiction of surface conservation

The evolution of protein surfaces and the conservation - or lack - thereof is another domain in which Structuprint can be applied. As an example, we performed a brief phylogenetic analysis of three orthologs of plastocyanin - a protein involved in electron transfer in photosynthesis [38] - for which crystallographic structures were available. The amino acid sequences of spinach plastocyanin (*Spinacia oleracea* [Swiss-Prot:P00289]) and those of two green algal species (*Ulva pertusa* [Swiss-Prot:P56274], *Ulva prolifera* [Swiss-Prot:P07465]) were retrieved from the UniProt database, along with the sequence of the spinach chloroplastic fructose 1,6-biphosphatase ([Swiss-Prot:P22418]) that would be later used as an outgroup. The sequences were aligned with ProbCons 1.12 [39] and the best model of amino acid substitution was determined with RAxML 8.1.16 [40]. Ten maximum likelihood trees were then inferred with RAxML using the biphosphatase as the outgroup sequence, and the best scoring tree was selected. Next, 2D protein surface maps of the corresponding 3D structures ([PDB:1AG6, 1IUZ, 7PCY, 1SPI]) were produced with Structuprint, after performing a structural superposition. For this example we used a more complex descriptor, FASA_H:

7 $$ FASA\_H = \frac{water\  accessible\  surface\  area\  of\  hydrophobic\  atoms}{water\  accessible\  surface\  area\  of\  all\  atoms} $$

**Fig. 4:**
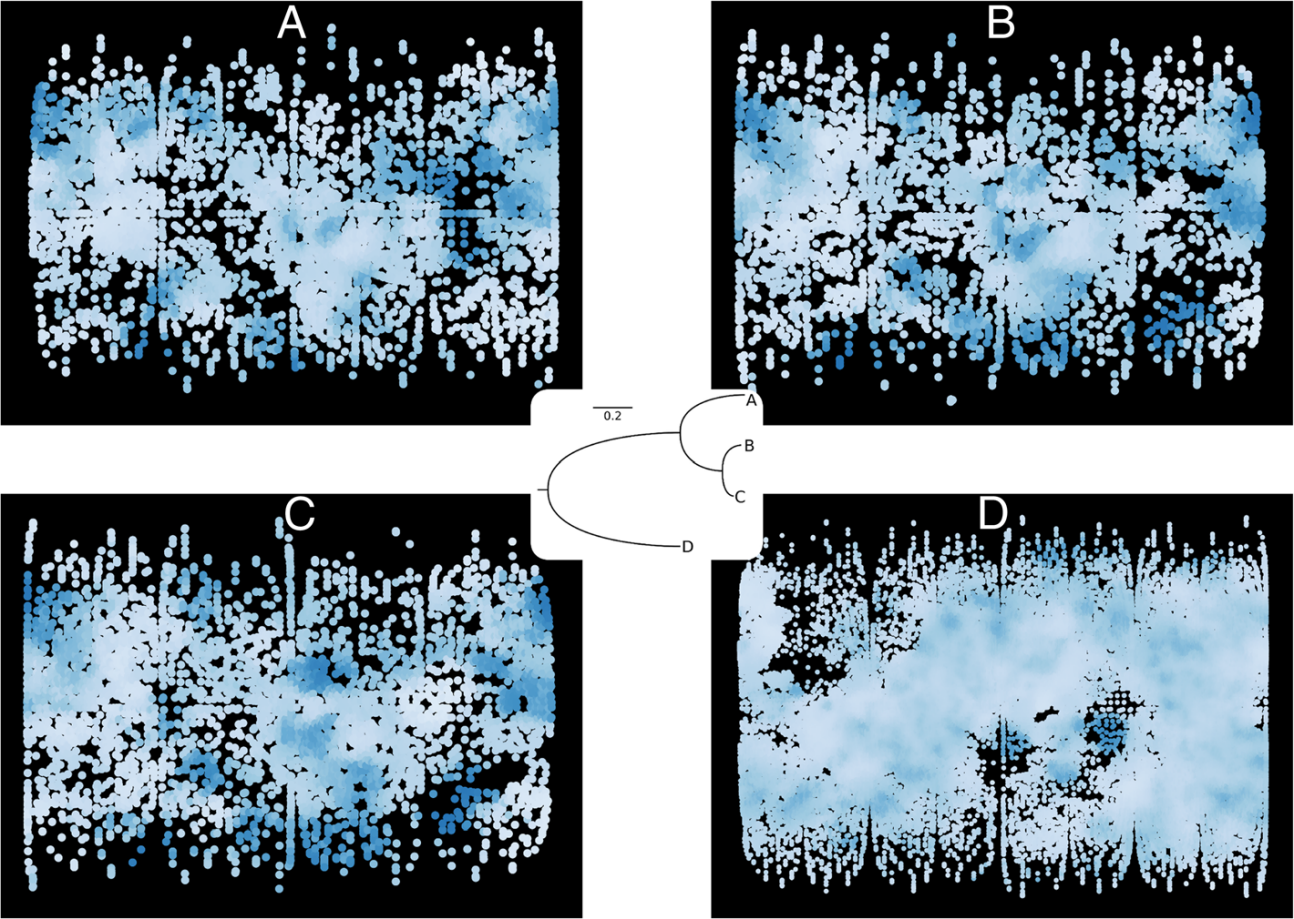
Evolution of protein surfaces, as represented via Structuprint figures. **a**–**c**: Plastocyanin orthologs from *Spinacia oleracea*, *Ulva pertusa*, and *Ulva prolifera*, respectively. **d** Chloroplastic fructose 1,6-biphosphatase from *Spinacia oleracea*. The colour depth denotes the FASA_H value across each map, with darker areas having higher values of the descriptor. Despite the obvious conservation of surface shape and hydrophobicity, 2D maps can distinguish even slight differences among evolutionarily related proteins. Inset: The maximum likelihood phylogenetic tree of the proteins in panels **a**–**d**

The results are shown in Fig. 4. There is significant conservation of both surface structure and hydrophobicity patterns among all three species, with the algal orthologs (Fig. 4b, c) exhibiting greater similarity, as expected. Finally, the representation of the chloroplastic fructose 1,6-biphosphatase (Fig. 4d) is vastly different from the others, highlighting the long sequence distance among these proteins.

#### Comparison of conformational changes, e.g., due to mutations

**Fig. 5:**
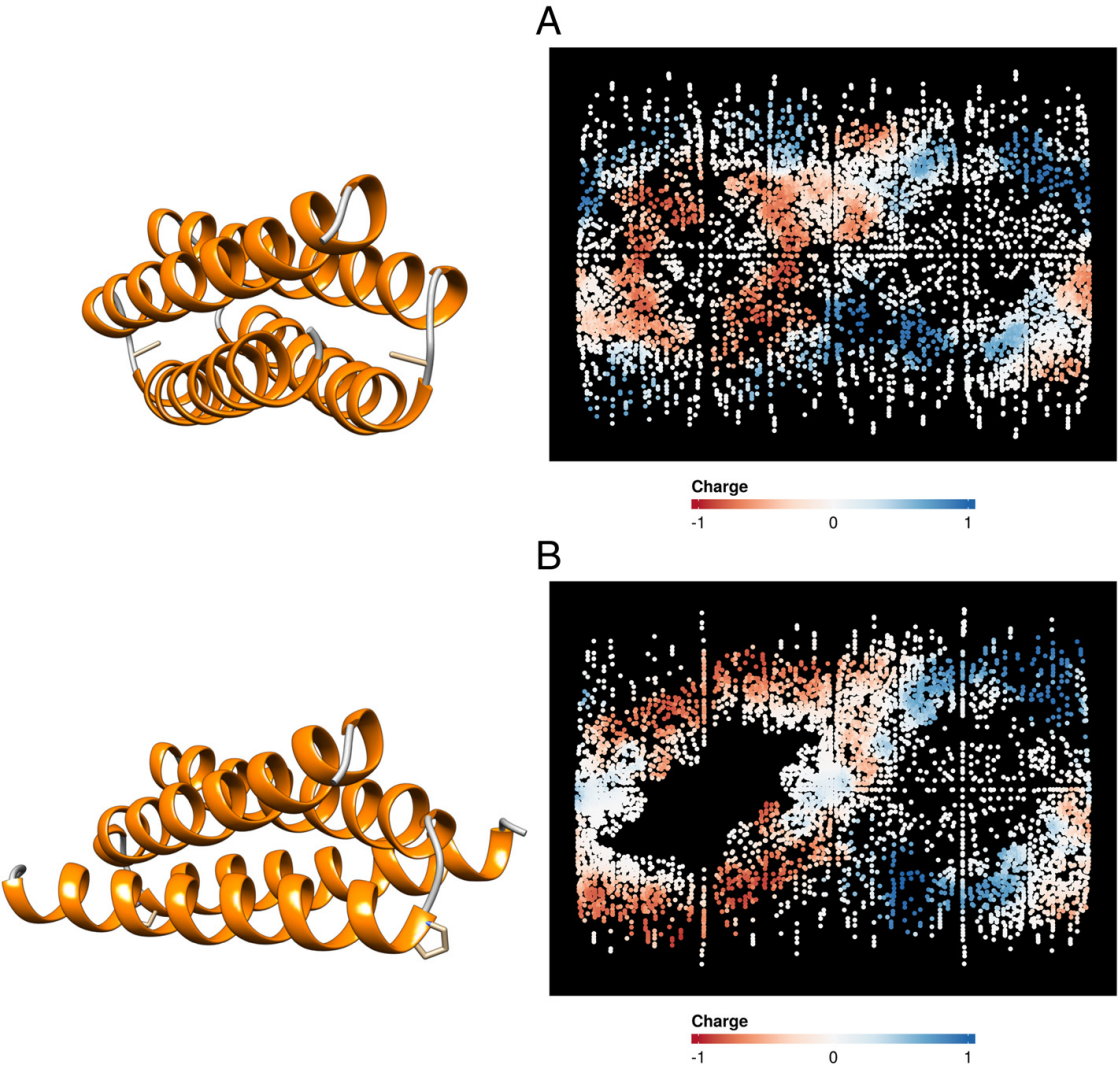
Three- and two-dimensional depiction of the native Rop structure (**a**) and the A31P mutant (**b**). In the 3D representation, the amino acid side chain of the 31st residue - in the turn region - is shown in stick style. Positively charged residues are shown with blue colour, negatively charged ones with red, and non-charged residues with white. With the 2D representation generated by Structuprint, large differences can be observed not only in the shape of the surface, but also in the location of exposed negatively charged residues

A third proposed application of Structuprint involves visually contrasting protein surfaces before and after events such as mutations, ligand binding, pH or temperature alterations. We exemplify this case using a mutant of Rop, a small regulatory protein from *Escherichia coli* with a native tertiary structure of a homodimeric four-helix bundle. The native structure has been shown to be disrupted by a single amino acid substitution (Ala31 → Pro) in the turn region [41]. To show the consequences of this mutation, we generated Structuprint maps of the wild type protein ([PDB:1ROP]) and the A31P mutant ([PDB:1B6Q]) after superposition. Figure 5 illustrates the mutation-induced conformation change, comprising different surface shape and grouping of negatively charged residues.

## Conclusions

We have developed a user-friendly application for two-dimensional visualization of protein surfaces, optionally supporting multicore processing and user-provided physicochemical descriptors. Structuprint provides an alternative view of molecular surfaces, which - as shown in the previous section - could be of great use to a variety of researchers, including biochemists, structural biologists, and biophysicists.

## Availability and requirements

**Project name:** Structuprint

**Project home page:**http://dgkontopoulos.github.io/Structuprint/

**Operating systems:** Prebuilt packages and installers are available for GNU/Linux distributions (Ubuntu 14.04, Debian 8, Fedora 22, CentOS 7, openSUSE 13.2), Windows, and OS X. For all other operating systems, installation from the source code is required. The GUI is available by default only for GNU/Linux systems.

**Programming languages:** Perl 5, R

**License:** GNU GPLv3+

**Any restrictions to use by non-academics:** None

## Availability of data and materials

The datasets supporting the conclusions of this article are included within the article and its additional files.

## Additional files

Additional file 1:
**Table of PDB entries used in the benchmark.** Accession codes and atom counts of 700 random, non-multimodel PDB entries that were included in the benchmark. (CSV 12 kb) Additional file 2:
**Conventional and molecular cartographic visualizations of a molecular dynamics simulation of the chicken villin headpiece subdomain (HP-35 NleNle).** Comparison between animations produced with conventional rendering methods (UCSF Chimera), and with 2D maps generated by Structuprint. The right half shows the movement of exposed amino acids with high topological polar surface area values (blue) during the course of the simulation. (GIF 4858 kb)

## Abbreviations

2D: two-dimensional
3D: three-dimensional
A31P: ala31 → pro mutant
CLI: command-line interface
CPU: central processing unit
FASA_H: fractional water accessible surface area of hydrophobic atoms over all atoms
GB: gigabyte
GUI: graphical user interface
HP-35 NleNle: villin headpiece subdomain double norleucine mutant (Lys24Nle/Lys29Nle)
MB: megabyte
MOE: molecular operating environment
MOPAC: molecular orbital package
PDB: protein data bank
RAM: random-access memory
RAxML: randomized axelerated maximum likelihood
