# Erratum to: Structuprint: a scalable and extensible tool for two-dimensional representation of protein surfaces

**DOI:** 10.1186/s12900-016-0057-5

**Published:** 2016-03-11

**Authors:** Dimitrios Georgios Kontopoulos, Dimitrios Vlachakis, Georgia Tsiliki, Sofia Kossida

**Affiliations:** Department of Life Sciences, Imperial College London, Silwood Park Campus, Ascot, UK; Bioinformatics & Medical Informatics Team, Biomedical Research Foundation, Academy of Athens, Athens, Greece; School of Chemical Engineering, National Technical University of Athens, Athens, Greece; IMGT®, The International ImMunoGeneTics Information System®, Université de Montpellier, Laboratoire d’ImmunoGénétique Moléculaire LIGM, UPR CNRS 1142, Institut de Génétique Humaine, Montpellier, France

Due to a formatting error during the production process of this article [1], equations 4 and 5 were published incorrectly in the PDF of the article [1]. The equations were, however, correctly presented in the HTML version of the article. Equations 4 and 5 are correctly included in this erratum.

The publisher takes full responsibility for the errors which occurred and sincerely apologises for the inconvenience caused.

4 $$ {\mathbf{w}}_{\boldsymbol{i}}=\left({x}_i^{\hbox{'}\hbox{'}},\ {y}_i^{\hbox{'}\hbox{'}},\ {z}_i^{\hbox{'}\hbox{'}}\right) = \frac{radius}{\sqrt{{x_i^{\hbox{'}}}^2+{y_i^{\hbox{'}}}^2+{z_i^{\hbox{'}}}^2}} \cdot {\mathbf{v}}_{\boldsymbol{i}}^{\boldsymbol{\hbox{'}}} $$

5 $$ \begin{array}{c}\hfill latitud{e}_i={ \tan}^{-1}\frac{z_i^{\hbox{'}\hbox{'}}}{\sqrt{{x_i^{\hbox{'}\hbox{'}}}^2+{y_i^{\hbox{'}\hbox{'}}}^2}}\ \hfill \\ {}\hfill longitud{e}_i={ \tan}^{-1}\frac{y_i^{\hbox{'}\hbox{'}}}{x_i^{\hbox{'}\hbox{'}}}\ \hfill \end{array} $$
