# Supplementary figures and images for: Structuprint: a scalable and extensible tool for two-dimensional representation of protein surfaces
Source: BMC Struct Biol. 2016 Feb 24;16:4. doi: 10.1186/s12900-016-0055-7 (PMC4765231; doi:10.1186/s12900-016-0055-7)

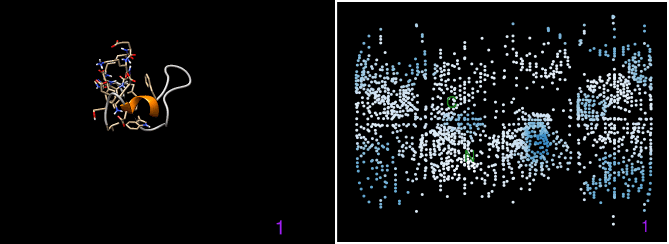

Supplement: Additional file 2: — Conventional and molecular cartographic visualizations of a molecular dynamics simulation of the chicken villin headpiece subdomain (HP-35 NleNle). Comparison between animations produced with conventional rendering methods (UCSF Chimera), and with 2D maps generated by Structuprint. The right half shows the movement of exposed amino acids with high topological polar surface area values (blue) during the course of the simulation. (GIF 4858 kb) [file 12900_2016_55_MOESM2_ESM.gif]
